# Geographical Access to Child and Family Healthcare Services and Hospitals for Africa-Born Migrants and Refugees in NSW, Australia; A Spatial Study

**DOI:** 10.3390/ijerph182413205

**Published:** 2021-12-15

**Authors:** Carolyne Njue, Nick Nicholas, Hamish Robertson, Angela Dawson

**Affiliations:** School of Public Health, Faculty of Health, University of Technology Sydney, P.O. Box 123, Ultimo, NSW 2007, Australia; nick.nicholas@uts.edu.au (N.N.); Hamish.Robertson@uts.edu.au (H.R.); angela.dawson@uts.edu.au (A.D.)

**Keywords:** spatial-access, healthcare, vulnerable populations, refugees, migrants, African, Australia

## Abstract

**Background:** African-born migrants and refugees arriving from fragile states and countries with political and economic challenges have unique health needs requiring tailored healthcare services and support. However, there is little investigation into the distribution of this population and their spatial access to healthcare in Australia. This paper reports on research that aimed to map the spatial distribution of Africa-born migrants from low and lower-middle-income countries (LLMICs) and refugees in New South Wales (NSW) and access to universal child and family health (CFH) services and hospitals. **Methods:** We analysed the Australian Bureau of Statistics 2016 Census data and Department of Social Services 2018 Settlement data. Using a Geographic Information System mapping software (Caliper Corporation. Newton, MA, USA), we applied data visualisation techniques to map the distribution of Africa-born migrants and refugees relative to CFH services and their travel distance to the nearest service. **Results:** Results indicate a spatial distribution of 51,709 migrants from LLMICs in Africa and 13,661 refugees from Africa live in NSW, with more than 70% of the total population residing in Sydney. The Africa-born migrant and refugee population in Sydney appear to be well served by CFH services and hospitals. However, there is a marked disparity between local government areas. For example, the local government areas of Blacktown and Canterbury-Bankstown, where the largest number of Africa-born migrants and refugees reside, have more uneven and widely dispersed services than those in Sydney’s inner suburbs. **Conclusion:** The place of residence and travel distance to services may present barriers to access to essential CFH services and hospitals for Africa-born refugees and migrants. Future analysis into spatial-access disadvantages is needed to identify how access to health services can be improved for refugees and migrants.

## 1. Introduction

International migration is driven by inadequate human and economic development, demographic increase and urbanisation, political and social factors, and environmental change is driving the world citizenry to live outside of their country of birth [1]. Australia is one of the top 10 popular destinations for migrants, with one third (29.8%) of her population born overseas [2]. More indicatively, except for Indigenous people, Australia is home to 25.7 million migrants or the descendants of migrants [3]. In the last decade, approximately 180,788 refugees have been resettled in Australia [4]. A migrant has been defined as “someone who makes a conscious choice to leave their country to seek a better life elsewhere [5]; and a refugee as “someone unable or unwilling to return to their country of origin owing to a well-founded fear of being persecuted for reasons of race, religion, nationality, membership of a particular social group, or political opinion” [6].

Africans are a relatively recent group of the increasing number of the overseas-born population migrating to Australia. African migrants and refugees increased from 7082 in 1997–1998 to 17,735 in 2004–2005 [7]. The latest reports to provide an overview of people born in Africa who have settled in Australia are based on the 2006 Census data [2,8] and identified 248,699 Africa-born migrants. Almost three quarters (72.6%) of this population were from Southern and East Africa, 22.9% originated from North Africa and 4.5% from Central and West Africa, and the majority of these arrivals are youthful and of reproductive age [2,8]. Research shows that African migrants and refugees can at times arrive with complex or multiple health problems [9,10]. Many migrants arrive with good English language skills, formal education and/or employment qualifications, but they face difficulties accessing health care services [11]. There is great diversity in access rights to health services; eligibility for government services, including migrant health services, depends on criteria such as visa subclass, period of residence in Australia, and the migrant’s circumstances [11]. Unlike migrants who have acquired permanent residence during their stay in Australia, no Medicare support is provided for temporary migrants; they have to purchase health insurance independently to access health services, including child and family health care and hospitals [12,13].

Refugees are particularly more disadvantaged, having fled their homes under traumatic circumstances to move to Australia [14]. The majority arrived with little or no English, have poor experiences accessing healthcare services, low health literacy affecting their communication with health providers, poor understanding of benefits and entitlements to access local health services and other social supports [14,15,16]. Complexities in visa types have led to confusion- while refugees under the humanitarian visas subclass 200–204 are entitled to government benefits, including the full range of healthcare services; for those under the special humanitarian visa subclass 202, eligibility for Medicare benefits is dependent on their sponsor for post-arrival assistance [17,18]. The lack of understanding of legal rights and entitlements may influence the uptake of publicly funded services, including primary healthcare services. Studies further show that refugees are often disproportionately represented among social security recipients and public housing tenants [19], and the cost of living in urban areas, including high unemployability rates, may influence their patterns of access to essential public and healthcare services and equity of health outcomes [14,20,21].

Access is an important determinant in the delivery and uptake of healthcare services [22,23] and may have a greater impact on the health of vulnerable populations [19], including Africans from refugee backgrounds. Public access to quality services is achieved when healthcare services are available and within reasonable reach of those who need them, acceptable, appropriate, cost-effective, and of good quality, irrespective of location and income [24,25]. Public access to quality services is central to Australia’s universal health care. The Australian healthcare system is jointly run by the federal, state and territory, and local governments who provide a wide range of healthcare services from population health and prevention to general practice and community health; emergency health services and hospital care; and rehabilitation and palliative care [26]. Primary health care is the first point of call for most people and is delivered in various settings, including general practices, community health centres, and homes. The universal health insurance scheme (Medicare) and the public hospital system provide free or low-cost access to health care for all Australian citizens and permanent residents. Private health insurance gives people a choice outside the public system. Health services in local areas are coordinated under primary health networks. The mix of services offered by an individual hospital varies according to the size of the local population and the services offered by other hospitals in the area.

Physical distance is measured by assessing where people are situated and the coverage of healthcare facilities at discrete location points [22]. Studies estimating the distances that people travel for regular healthcare services, retail and work, suggest that the proximity of households, on average, should ideally be within 5 km distance to a healthcare service [27,28]. The WHO states that health services should be conveniently and efficiently placed to serve and monitor the communities [29]. Spatial access to healthcare remains a global concern, with the most remote and rural areas experiencing poorer access than more populated and urban areas. In Australia, population growth is more concentrated in cities or the coast where access to public and private services systems tends to be located [22,30]. Mobility, health services, and the distribution of vulnerable populations in urban areas of Australia have not been fully explored in research and policy domains, as health access is typically assumed not to be a significant problem because of this and the growth of the public transport system and high levels of car ownership [24,31].

Access to health services is typically framed by the distribution of health services and ignores inter-locality problems of collective public transportation policies [32]. Moreover, mobility inequality, defined as disadvantages to individuals and groups produced by unequal access to resources for physical accessibility, is best understood in its socio-spatial context [33]. A detailed analysis of the geography of relative collective transport deprivation (hardship) can demonstrate that various populations experience different health outcomes that may be related to mobility inequalities due to the distinctive geography of Australia [24,34]. According to Giorgi and Vitale, the evolution of mobility inequalities for undocumented migrants and refugees has become an issue in the public discourse in different countries [34]. Pratschke and colleagues identified study measures and results concerning the relation between drive distance from healthcare services (and other welfare services) and the feeling of disenfranchisement of individuals and families living in peripheral urban areas. They found an association between residential localisation, driving distance and exposure to health opportunities [32]. The geography of mobility is pivotal to understanding health inequalities. Therefore, further geographical linkage to health data can allow us to chart the different distance/mobility outcomes experienced by the different distance-based groups, such as the Africans from refugee backgrounds living in urban Australia.

In this study, we update the settlement patterns of Africa-born refugees and migrants from low and lower-middle-income countries living in New South Wales (NSW) urban contexts and examine the distribution of this population in relation to their travel distance to public child and family health (CFH) services and hospitals.

## 2. Materials and Methods

The study population included migrants from only low and lower-middle-income countries (LLMICs) of Africa and all African refugees. We excluded from the analysis all migrants from high-income and upper-middle-income African countries (i.e., with a gross national income (GNI) per capita of between $3996 and $12,375) [35]. This includes migrants from countries such as South Africa, which represented 42% (*n* = 388,179) of the total population of African migrants living in Australia [2], Equatorial Guinea, Algeria, Libya, Gabon, South Africa, Botswana, Mauritius, Namibia, and Seychelles, as they may not fit our criteria for study population groups. Only migrants from countries considered as low (economies with a GNI per capita of $1025 or less in 2018) and lower-income (economies with a GNI per capita between $1026 to $3995) [35] countries were included given their circumstances, vulnerabilities and specific access needs. 

This study focused on NSW, which is the most populous state in Australia. 

### 2.1. Data Frames

Population data of migrants from African LLMICs were obtained from the Australian Bureau of Statistics (ABS) 2016 Census of Population and Housing data, using their country of birth (COB) [2]. To supplement this, we used the Department of Social Services (DSS) Settlement data to identify the refugee category using indicators such as African countries of birth (COB) and visa category [36]. The inclusion of both datasets is important to identify settlement patterns and needs specific to humanitarian or non-humanitarian stream populations. The following variables were examined: country of birth, socio-demographic characteristics, visa category, languages are spoken, residential addresses and socio-economic status (SES) postcode measures. These datasets were downloaded from the ABS website using TableBuilderPro (i.e., the census data) and analysed directly from the DSS settlement database (i.e., for refugee data) (Appendix A). 

### 2.2. Service Location Data

We compiled a broad list of CFH services and hospitals in NSW in an Excel spreadsheet, identifying each service’s location and postal addresses/postal-code information. The addresses of the health services were obtained from various open sources, including the NSW Government health services directory, websites [37,38], yellow pages and other planning documents [39,40]. We focused on universal CFH services [41]. Hospitals were included as a complementary set of physical reference points.

We mapped the geographic differences in the distribution of Africa-born migrants within NSW according to the smaller ‘statistical area 2’ (SA2) categories to estimate population coverage in various catchment areas. SA2 is a medium-sized geographical area with populations between 3000 and 25,000 persons that can facilitate the identification of socio-economic patterns within the African communities [42]. All the target population households were geocoded at the statistical-area level 2 (SA2) to ensure this population group’s confidentiality, many of whom could potentially be identified at the SA1 mapping level. However, we were unable to geocode 62 (0.1%) migrant individuals who did not have fixed or defined addresses as their place of residence using a GIS process. As a result, these persons were excluded from the analysis. The CFH services were also added as separate layers onto a mapping system and overlaid onto the SA2 base allowing us to map the numbers and distribution by service type. 

Spatial analysis was undertaken using Maptitude GIS mapping software to physically examine map data [38]. All the data sets were imported into Maptitude for consistency in the analysis phase and to permit multiple spatial analysis tools to be applied, including converting the postal address/postal-code information into a geotagged set of cross-sectional data and measuring the population density and access to health services. We assumed that travelling to the nearest healthcare service would most likely be undertaken by private car or public transit given the region’s public and private transport mode.

Travel distance was calculated in kilometres (km). We obtained information on health service location and the travel distance, adopting the 5 km radius population grid estimate with cut-off points at the 5 km, 10 km and 20 km travel distance of healthcare service. These cut-off points are based on previous studies that have estimated the distances people travel for regular healthcare services, retail and work [27,28]. This model also assumes that patients may, first and foremost, seek hospital care within the catchment area and local health districts (LHDs) where people live, and therefore, a healthcare service should be conveniently and efficiently placed to serve them [27,29]. There are eight LHDs covering the metropolitan regions. Ethical approval was not required for this study, as all data is available in the public domain.

## 3. Results

### 3.1. Profile of Africa-Born Migrants and Refugees

Approximately 27% (*n* = 51,709) of migrants from African LLMICs live in NSW (2) (Table 1). Additionally, humanitarian entrants’ data from the Department of Social Services Settlement (DSS) database showed that 19% (*n* = 13,661) of all refugees from African countries recorded in Australia are residing in NSW [36] (Table 2). This number includes only those Africans possessing a refugee category visa (i.e., subclasses 200–204 and 866). The proportion between males (52%) and females (48%) is relatively similar.

According to the 2016 Census, the top ten countries of birth for the Africa-born LLMIC migrants in NSW were Egypt, Zimbabwe, Sudan, Kenya, Ethiopia, Nigeria, South Sudan, Somali, Zambia and Ghana. The DSS 2018 settlement data shows that refugees from African countries came mainly from fragile and post-conflict countries such as Sudan, the Democratic Republic of Congo and Ethiopia, Egypt, Sierra Leone, Liberia, Somali, Kenya, Eritrea and Burundi. The majority (78.1%) of the African migrants from LLMICs were in the economically active age groups (between 16–64 years), with a median age of 39 years. Approximately two out of ten Africa-born migrants (23.7%) had completed high school education (Year 10 and above), compared to more than half (52.7%) of the general population in NSW aged 15 and over, who held a post-school qualification [2]. 

There were more male (54%) than female (46%) refugees. Approximately 61% of the refugee population in NSW were in the working-age group of 16–64 years, 38% were children aged 0–15 years, and one percent were persons over the age of 65 years. About 43% of the refugees did not speak English on arrival, 31% reported having poor English skills, and only 9% and 5% respectively said their English was “good” or “very good”.

### 3.2. Geographical Settlement

**Figure 1:**
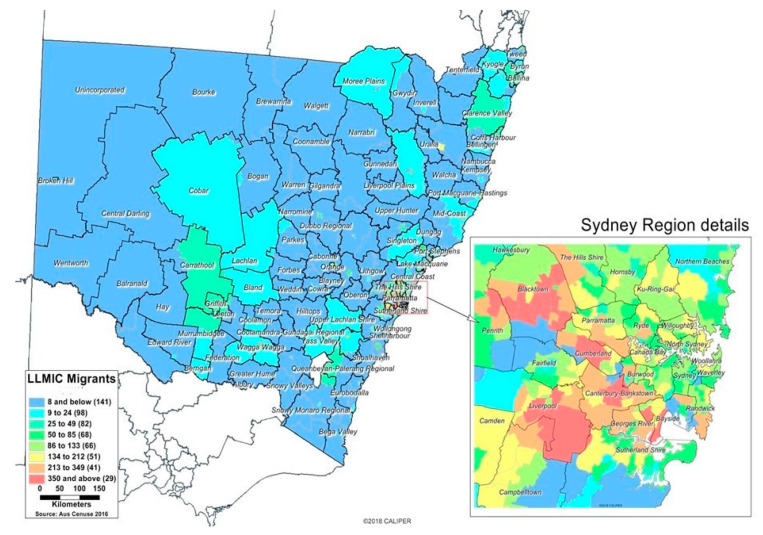
The spatial distribution of Africans from LLMICs in NSW at the statistical-area level 2 (SA2) level, ABS Census data 2016. In the map legend, numbers in parentheses are a count of SA2 areas and those without the migrant population. The inset map is a close-up of the spatial distribution of these population groups in metropolitan areas.

**Figure 2:**
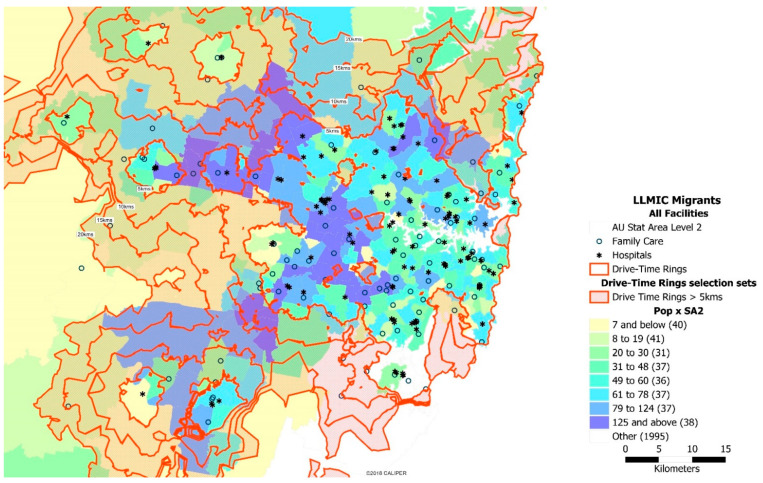
The spatial distribution of migrants from LLMIC and refugee communities relative to a CFH service and/or hospital and their drive distance most travel to access care. The dark coloured areas indicate where most of them live, while the white dots represent the CFH services. Our approach defines travel time using area contours increasing incrementally starting at 5 km travel distance; that means that the inner contour is within 5 km of healthcare service. All areas outside the 5 km zone are shaded, whilst those within a 5 km access level are not shaded. The loops show travel distances within those areas.

Over 70% of Africans lived in the state capital, Sydney, mostly in the outer-urban areas of Western Sydney (Figure 1 and Figure 2). The local government areas of Blacktown (6561 migrants, 6627 refugees), Canterbury-Bankstown (6043 migrants, 3489 refugees) and Liverpool (3387 migrants, 3466 refugees) were identified as having the highest proportion of the African population. Most migrants from LLMICs and refugee populations in regional and rural areas of NSW live in Wagga Wagga and Coffs-Harbour’s local government areas. Overall, less than one percent of the migrants from LLMIC and the refugee population in NSW reside in remote and very remote areas. 

### 3.3. CFH Services and Travel Distance

The distribution of migrants from LLMIC and refugee communities relative to the nearest CFH services and their travel distance across these metropolitan areas was further explored. There is variation in the spatial distribution of CFH services between inner and outer Sydney areas (Figure 2). For example, the distribution of CFH services appears unevenly and widely dispersed in the LGAs of Blacktown and Canterbury-Bankstown, notable areas where the highest number of African-born migrants and refugees in NSW reside. 

**Figure 3:**
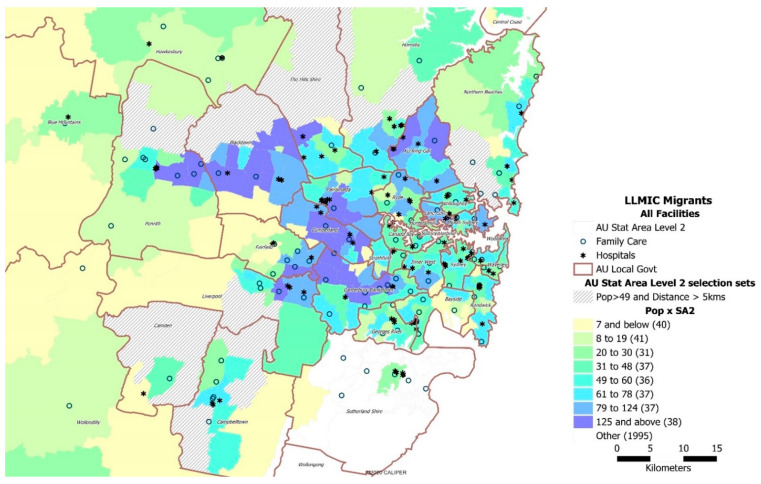
The spatial distribution of migrants from LLMICs and refugee communities relative to a CFH service. The vertically striped greyed areas show SA2 areas where the African population is greater than 49 people and pockets of residential areas where the nearest healthcare service is further than a 5 km drive.

African migrants from LLMICs and refugee communities in Sydney are generally well served by CFH services, based on the 5 km travel distance and proximity to the healthcare services (Figure 3). However, there is a marked disparity between local government areas. The CFH services in the Blacktown and Canterbury-Bankstown LGAs, where the largest number of migrants and refugee communities reside, are more unevenly and widely dispersed than those in Sydney’s inner suburbs. Moreover, there are 99 metropolitan-fringe suburbs outside the 5 km zone that could be identified as ‘access-disadvantaged’ areas, given their distances to the nearest CFH service.

## 4. Discussion

This study has provided insight into the spatial distribution of African migrants from LLMICs and refugee communities in NSW and travel distance to CFH services and hospitals. Approximately 27% of migrants from LLMICs and 20% of the African refugee population in Australia live in NSW, with the majority in Sydney, more specifically in the Greater Western Sydney metropolitan areas. The Greater Western Sydney metropolitan areas appear to be well served by CFH services. However, the more unevenly and widely dispersed CFH services in Blacktown and Canterbury-Bankstown LGAs may particularly affect African women, who are the main carers for children and [14], may be required to travel with children to health care appointments [31]. This may be magnified in settings where women lack family networks or support [43,44]. Gender differences have also been noted to have different effects on psychological health [45]. Women’s mental health appears to be more negatively affected by longer commute times than men due to their household duties and caring responsibilities [14,44,45]. 

African refugee and migrant communities living in rural and remote areas of NSW may be particularly disadvantaged by the longer travel distances required to reach some of the targeted or specialist health services [24,30,31]. This may need to be examined in light of the increasing focus on resettling new African arrivals in regional areas, including Coffs Harbour, Goulburn Wollongong, Wagga Wagga and Newcastle. These findings could be interpreted from a structural and organisational point of view to delivering healthcare, given the way in which they intersect with multiple factors, including individuals’ legal status, resettlement, length of stay, and stress accessing healthcare resources. Studies have found that remoteness of a settlement, perceived discrimination in terms of benefits and healthcare entitlements, accessing resources, including social and language supports, public transport and employment, might worsen healthcare provision for people from refugee backgrounds [18,46,47]. According to Ziersch and colleagues, discrimination has been attributed to adverse impacts on the mental health of people from refugee backgrounds in Australia [46]. King and colleagues also argue that (1) compulsory assigned residency, (2) resources (included language skills), and (3) freedom of movements (in our case, related to visa category restrictions) could be consolidating heavy and stable forms of devaluation, reification, and stigma, denying the access to healthcare for certain groups of migrants and refugees in Europe [48].

The NSW government has made universal CFH services more responsive to African community needs. Bi-lingual workers, maternity liaison officers and community-based workers have been employed through the government’s multicultural health services program and by LGAs to support African communities access healthcare, interpreters and link them to English language education and social networks [49,50]. The Department of Social Services Settlement Grants Program also supports refugees for the first five years after arrival, facilitating access to English language services, public transport concessions, kindergarten and childcare fee subsidies, education and training, employment, and housing [17]. In addition, the NSW Refugee Health Service (RHS) operates general practice clinics in Western Sydney. These one-stop-shop clinics offer health assessments, referrals to outpatient clinics and other services (e.g., dental clinics, women’s health) and health education materials for refugees [51]. 

Mainstream healthcare for refugees and migrants is complemented by non-governmental organizations (NGOs. Non-profit organisations and community agencies such as the Refugee Council of Australia provide information on and advocacy for refugee and humanitarian entrants [52]. The Migrant Resource Centres and Settlement Services International assists newly arrived migrants, refugees, and humanitarian entrants in their settlement process. The agencies work closely with local and regional service providers, including primary health centre facilities, practitioners and community groups, to improve screening, culturally appropriate referrals, navigation through health, refugee trauma and settlement issues, access to employment, housing, health literacy, nutrition, mental health, child protection, foster care, youth transition, disability support services [53,54]. They employ multicultural support workers who promote culture-specific programs, activities and resources to enhance service awareness, improve access, equity and participation of Australia’s growing culturally and linguistically diverse (CALD) populations, including refugees [50].

However, while all these support services, including the provision of tailored education materials, are essential, there is a need to involve the consumers in the design of community projects so that access issues can be fully understood and services tailored to meet their needs in ways that are acceptable to them [50,55]. For instance, there is a need for community development programs that improve social capital to promote social support, social adjustment, social inclusion and active citizenship [56,57]. Moreover, public transportation insecurity, including concerns of gender-based violence against African women, have been raised by the Media in Australia [58], and including unreliable or poorly linked bus transportation, have emerged as key reasons women miss or delay accessing the vital care they need, calling for the need to provide convenient transport programs and healthcare options. Transportation programs, including non-emergency medical transportation models being piloted in different settings in partnership with the private sector using ride-hailing companies like Uber and Lyft, can offer solutions to get migrant and refugee women to and from their healthcare appointments [24,59]. 

### Study Limitations

This study’s key limitation is the lack of included data on general practitioners’ (GP) location, who play a crucial role in Australia’s primary care services. Data on GPs is incomplete and presents categorisation challenges. The study shares methodological limitations with refugee and migrant datasets [60]. These datasets do not permit analysis to provide insight into access to healthcare services at an individual level to describe dimensions of vulnerability, such as mobility, social isolation, or literacy. The refugee data was also based on the settler’s latest known residential (or intended residential) address at the LGA level data. Our study indicates the need for population-specific surveys to enable analysis at the individual level within neighbourhoods or urban and rural regions. Additionally, our drive-time analysis to assess spatial accessibility to healthcare services within metropolitan settings assumed that individuals prefer care services close to home and would only travel further if services close by did not meet their needs. Given our assumptions, these results may not readily translate to other geographical settings outside this region.

## 5. Conclusions

This study contributes to demographic and public health research and provides insights into the spatial distribution of migrants from LLMICs and refugee communities in NSW and their access to CFH services and hospitals. Travel distance may present a barrier to this population’s healthcare utilisation in LGAs, where healthcare services are more widely dispersed. Acknowledging travel distance as a determinant of health will enable consideration of the importance of safe public transport and local healthcare service provision. Future research is needed to examine disadvantages related to geographical access to health services to provide an accurate picture of challenges and opportunities to inform urban and health service planning.

## Figures and Tables

**Table 1:** Characteristics of the Africa-born migrant population.

Variables	Frequency (%)
All Africa-born migrants in Australia Originate from South Africa	388,179 162,705 (42)
Africa-born migrants LLMICS Originate from LICs Originate from LMICs	191,876 91,951 (48) 99,925 (52)
Gender Male Female	99,776 (52) 92,100 (48)
Age Aged < 16 Persons aged 16 to 64 Persons aged 65+	31,449 (8.1) 307,820 (79.4) 48,911 (12.6)
Marital Status Married Never married Widowed Divorced Separated Not stated	(53) (25.3) (3.6) (6.5) (3.5) (8.1)
Education Primary Secondary College/University Not stated	(4.2) (38.6) (48.5) (8.7)
Income level (weekly) Low $0–$999 Middle $1000–$1999 High $2000 and above Not stated	(61.4) (22.7) (12.1) (3.0)
Residents by State New South Wales Victoria Queensland Western Australia South Australia Australia Capital Territory Tasmania Northern territory	51,709 (26.9) 52,150 (27.2) 31,019 (16.2) 36,329 (19) 12,991 (6.8) 3677 (2) 1983 (1) 1982 (1)

Source: Australian Bureau of Statistics (ABS). Census of Population and Housing, 2016.

**Table 2:** Characteristics of refugees from Africa (2000–2018).

Variables	Frequency (%)
Refugees by State New South Wales Victoria Queensland Western Australia South Australia Australia Capital Territory Tasmania Northern territory External territories	(*n* = 70,637) 13,661 (19.3) 21,409 (30.3) 14,526 (20.5) 9500 (13.4) 7483 (11) 1022 (1.4) 1772 (2.5) 1064 (1.5) 100 (0.1)
Refugee visa category Subclass 200 Subclass 201 Subclass 202 Subclass 203 Subclass 204 Subclass 866	28,539 (40) 541 (0.8) 30,180 (43) 58 (0.1) 5974 (8.5) 5345 (7.6)
Gender Male Female	37,830 (54) 32,807 (46)
Age Aged < 15 Persons aged 16 to 64 Persons aged 65+	27,246 (38) 42,925 (61) 466 (1)
Marital Status Engaged/Defacto partner Married Never married Separated/Divorced Widowed Not stated	971 (1.4) 16,586 (23.5) 25,684 (36.4) 1280 (1.8) 2153 (3) 23,963 (33.9)
Proficiency in English Nil Poor Good Very good Not stated	30,521 (43) 21,940 (31) 6206 (9) 3854 (6) 8116 (11)

Source: Department of Social Services, Settlement Reporting June 2018.

## Data Availability

Not applicable.

## Abbreviations

ABS—Australian Bureau of Statistics

ACT—Australian Capital Territory

CFH—Child and Family Health

DSS—Department of Social Services

GIS—Geographic Information System

GP—General Practitioner

LGA—Local Government Area

LLMIC—low and lower-middle-income countries

NSW—New South Wales

NGO—Non-governmental organisation

RHS—Refugee Health Service

WHO—World Health Organisation

## Appendix A. Data Sources-Availability of Data and Materials

Australian Bureau of Statistics (ABS). *Census of Population and Housing, 2016.* Available from: https://www.abs.gov.au/AUSSTATS/. using TableBuilderPro (accessed on 10 January 2019).

DSS. Settlement Reporting: Department of Social Services, December 2018. Available from: https://www.dss.gov.au/our-responsibilities/settlement-services/programs-policy/settlement-services/settlement-reporting (accessed on 30 April 2020).
